# Habitat, wildlife, and one health: *Arcanobacterium pyogenes* in Maryland and Upper Eastern Shore white-tailed deer populations

**DOI:** 10.3402/iee.v3i0.19175

**Published:** 2013-08-06

**Authors:** Melissa M. Turner, Christopher S. DePerno, Mark C. Conner, T. Brian Eyler, Richard A. Lancia, Robert W. Klaver, Michael K. Stoskopf

**Affiliations:** 1 Department of Forestry and Environmental Resources, Fisheries, Wildlife, and Conservation Biology Program, North Carolina State University, Raleigh, NC, USA; 2 Chesapeake Farms, Chestertown, MD, USA; 3 Department of Natural Resources, Annapolis, MD, USA; 4 United States Geological Survey, Sioux Falls, SD, USA; 5 Environmental Medicine Consortium and Department of Clinical Sciences, College of Veterinary Medicine, North Carolina State University, Raleigh, NC, USA

**Keywords:** Arcanobacterium pyogenes, white-tailed deer, one health, intracranial abscessation, emerging diseases

## Abstract

**Background:**

Understanding the distribution of disease in wildlife is key to predicting the impact of emerging zoonotic one health concerns, especially for wildlife species with extensive human and livestock interfaces. The widespread distribution and complex interactions of white**-**tailed deer (*Odocoileus virginianus*) with humans suggest deer population health and management may have implications beyond stewardship of the animals. The intracranial abscessation suppurative meningitis (IASM) disease complex in deer has been linked to *Arcanobacterium pyogenes*, an under**-**diagnosed and often misdiagnosed organism considered commensal in domestic livestock but associated with serious disease in numerous species, including humans.

**Methods:**

Our study used standard bacterial culture techniques to assess *A. pyogenes* prevalence among male deer sampled across six physiogeographic regions in Maryland and male and female deer in the Upper Eastern Shore under Traditional Deer Management (TDM) and Quality Deer Management (QDM), a management protocol that alters population demographics in favor of older male deer. Samples were collected from antler pedicles for males, the top of the head where pedicles would be if present for females, or the whole dorsal frontal area of the head for neonates. We collected nasal samples from all animals by swabbing the nasopharyngeal membranes. A gram stain and catalase test were conducted, and aerobic bacteria were identified to genus and species when possible. We evaluated the effect of region on whether deer carried *A. pyogenes* using Pearson's chi-square test with Yates’ continuity correction. For the white-tailed deer management study, we tested whether site, age class and sex predisposed animals to carrying *A. pyogenes* using binary logistic regression.

**Results:**

*A. pyogenes* was detected on deer in three of the six regions studied, and was common in only one region, the Upper Eastern Shore. In the Upper Eastern Shore, 45% and 66% of antler and nasal swabs from deer were positive for *A. pyogenes, respectively*. On the Upper Eastern Shore, prevalence of *A. pyogenes* cultured from deer did not differ between management areas, and was abundant among both sexes and across all age classes. No *A. pyogenes* was cultured from a small sample of neonates.

**Conclusion:**

Our study indicates *A. pyogenes* may be carried widely among white-tailed deer regardless of sex or age class, but we found no evidence the pathogen is acquired in utero. The distribution of *A. pyogenes* across regions and concentration in a region with low livestock levels suggests the potential for localized endemicity of the organism and the possibility that deer may serve as a maintenance reservoir for an emerging one health concern.

The ‘one health’ concept is enjoying resurgence as physicians, scientists, and veterinarians develop greater appreciation for the health implications of the complex interactions between the environment, humans, and domestic and wild animals (1–3). Wildlife species serve important roles in one health processes as key players in disease interactions involving feral and domestic livestock, and through direct impacts on human health. The majority of emerging infectious diseases in humans are zoonotic (4), and most of these are believed to originate from wildlife populations or to be amplified though interactions between wildlife and domestic and feral livestock (5, 6). Understanding the spatiotemporal distributions of disease in wildlife is key to identifying the dynamics and predicting the establishment of emerging infectious diseases (7).

White**-**tailed deer *(Odocoileus virginianus*) represent one of the most abundant and widely distributed large ruminant mammal species in North America (8–10). In the United States, white**-**tailed deer populations have expanded vastly since the early 20th century through management focused on species recovery (11, 12). Human**–**deer and deer**–**livestock interactions have increased and evolved with the expansion of the interface boundary among the species, though more quantitative data on this phenomenon are needed (13, 14). Approaches to management of burgeoning deer populations have implications beyond resource stewardship, including the potential for increased deer**–**human conflict (15) and for deer populations to serve as reservoirs for infectious diseases (5). Similarly, environmental impacts on pathogen viability in different physiogeographic environments can alter the overall impact of potential emerging one health concerns.

The intracranial abscessation suppurative meningitis disease complex (IASM) is generally considered a minor cause of population**-**wide mortality among white**-**tailed deer in the United States (16, 17) However, infection prevalence differs regionally, and the disease complex can contribute substantially to deer mortality in some areas. IASM has been associated with high losses on the Upper Eastern Shore of Maryland, where it was pinpointed as the cause of nearly 35% of annual mortality among mature males (18). Skulls of animals affected by IASM are characterized by erosion and pitting of bones, and the frequent occurrence of fluid**-**filled nodules beneath the antler pedicle, lesions that are not readily appreciated in living deer (Fig. 1). Clinically, antlers may be disfigured and the antler pedicle is typically surrounded by inflamed tissue and extravasated viscous fluid (Fig. 1). Clinical signs of IASM in white-tailed deer mimic other key zoonoses and include incoordination, lack of fear, blindness, weakness, emaciation, and circling (16, 19).

**Fig. 1:**
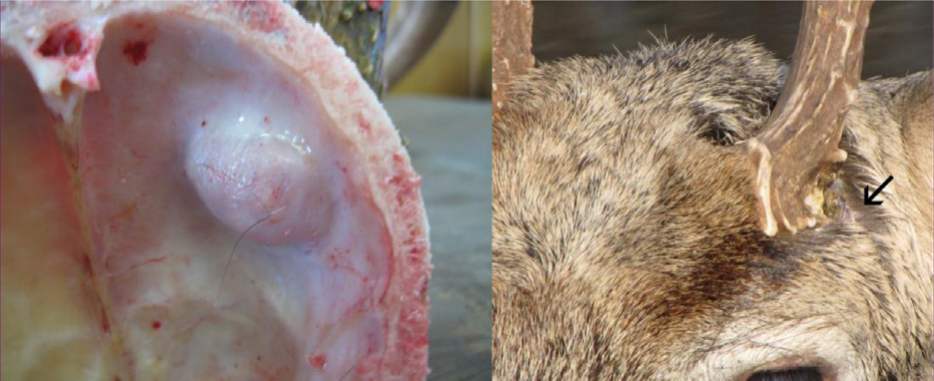
Pitting of bone and fluid-filled nodule inside skull plate of infected white-tailed deer and male white-tailed deer with signs of IASM at Chesapeake Farms, Maryland, USA.

The precise etiology of IASM in white**-**tailed deer is not definitively established, and though other bacteria can be cultured from lesions meeting the criteria of the syndrome, studies to date have shown that abscesses are most frequently associated with the gram**-**positive, non**-**motile, non**-**spore**-**forming, short, rod**-**shaped bacterium *Arcanobacterium pyogenes* (formerly *Corynebacterium* and *Actinomyces 1982*) (16, 17). *A. pyogenes* is generally considered a commensal organism and an opportunistic pathogen of domestic livestock, particularly cattle (*Bos* *primigenius*) and swine (*Sus* *scrofa*). It is a common inhabitant of the mucous membranes of both cattle and swine and can be routinely isolated from the digestive tract, udders, urogenital region, and upper respiratory tracts of healthy animals (20–25). The organism has also been isolated from clinical infections in a wide range of domesticated and wild ungulates, including domestic sheep (*Ovis aries*), blackbuck (*Antilope cervicapra*), and fallow deer (*Dama dama*) (24–31). *A. pyogenes* has been associated with various disease conditions ranging from abortion to osteomyelitis (21, 32). It is possible that *A. pyogenes* plays a similar role in white**-**tailed deer populations, which could serve as a reservoir for the pathogen in the absence of abundant livestock.

*A. pyogenes* expresses several known and suspected virulence factors, which may explain its ability to colonize many different host tissues and cause a diverse range of diseases (29). *A. pyogenes* is not considered part of the normal human flora (29), and it is an under**-**recognized and frequently misdiagnosed human pathogen with the potential to serve as a primary pathogen, though it is more commonly isolated as part of a mixed infection (33, 34). Under reporting of *A. pyogenes* infections likely occurs because the organism's biochemical profile is very similar to that of *A. haemolyticum* (34, 35). Many but not all human cases of *A. pyogenes*-related disease reported in the literature have been associated with underlying health problems including diabetes and cancer (36), but the organism can express a wide range of virulence factors, and the pathogenesis of infection by *A. pyogenes* is not well characterized (29). The broad array of human disease conditions reported due to *A. pyogenes* includes abdominal abscessation, otitis media, cystitis, mastoiditis, septicemia, sigmoiditis, appendicitis, cholecystitis, peritonitis, endocarditis, meningitis, arthritis, empyema, and pneumonia (34, 35, 37–41). Some authors suggest the predominant risk factors associated with human disease include close contact with animals, but that contact is not always recognized in the history or signalment of the specific cases (34). We believe this contributes to the lack of published reports of human cases associated with deer contact, and we are unaware of any aspect of the understood pathogenesis and transmission routes from domestic animals that would preclude infection from exposure to deer. In addition, hunting brings humans and deer into close contact providing a possible route of *A. pyogenes* transmission between IASM**-**infected animals as well as animals serving as a reservoir for the pathogen.

The *A. pyogenes* **-**associated IASM syndrome in white**-**tailed deer presents unique opportunities to examine factors that may play a role in the dynamics of what may be an under**-**recognized emerging one health problem. Adult male white**-**tailed deer appear to be particularly susceptible to IASM (16, 18), which could be an important mortality factor in deer populations operated under management strategies that foster a balanced sex ratio and an older age structure among male deer such as quality deer management (QDM) (42). If strategies such as QDM contribute to IASM, management decisions may have broader impacts on the health of the deer population than previously recognized. Management decisions for deer may impact the health of feral and domestic livestock as well as the health of humans who come in close contact with deer, for example during hunting season. Infections in male deer are more frequently recognized, but female deer and fawns (>6 months old) can acquire IASM and pulmonary, mammary, and disseminated systemic infections associated with by *A. pyogenes* (17, 43, 44). These health outcomes suggest deer management strategies might not be the only determinant of the prevalence of the disease complex. Other factors such as environmental characteristics and livestock prevalence may play a role in the impact of the bacterium. Though *Arcanobacterium* infections and cervid IASM occur across much of North America (17), there is evidence that *A. pyogenes* may not thrive under certain environmental conditions, particularly in arid climates (17, 18).

In the context of data indicating high prevalence of IASM in some deer populations as well as our hypothesis that *A. pyogenes* might play a similar role in deer populations as it does in other ungulate populations, we initiated a study on the prevalence of *A. pyogenes* across Maryland. We used recognized long-standing and well-accepted bacterial culture and biochemistry identification techniques to assess the prevalence of *A. pyogenes* across deer populations in different physiogeographic regions and under different deer management strategies to evaluate the impacts of these factors on a potential emerging one health concern.

## Ethics statement

All procedures followed guidelines set by the North Carolina State University Institutional Animal Care and Use Committee (09**-**065**-**O), and all animal handling methods used followed guidelines approved by the American Society of Mammalogists (45). The Maryland Department of Natural Resources provided permission for all studies conducted for this project, including live capture of white**-**tailed deer fawns and sampling from hunter**-**killed adult white**-**tailed deer in the Upper Eastern Shore, Lower Eastern Shore, Western Shore, Piedmont, Ridge and Valley, and Appalachian provinces of Maryland.

## Methods

Three related studies were conducted. In all studies, we determined sex of white**-**tailed deer by manual palpation and visual examination and assigned to age classes (i.e. 0.5, 1.5, and 2.5+) using tooth replacement and wear (46). Animals were weighed when possible and examined physically for external visible signs of disease or injury. We used successful culture of viable bacteria, a highly discriminatory approach for detection of the presence of *A. pyogenes* as our diagnostic standard. Following the methods of Karns et al. (18), we collected head and nasal swabs from each animal using Remel Bacti**-**Swab transport swabs (Thermo Fisher Scientific, Waltham, Massachusetts, USA). For head samples, we swabbed around antler pedicles for males, and residue was collected from the top of the head where pedicles would be, if present for females, or the whole dorsal frontal area of the head for neonates. We collected nasal samples by swabbing the nasopharyngeal membranes by inserting the sterile swab deep into the nasal cavity of one nostril taking care not to contact the external nares, and rotating the swab gently before removing it. All samples were kept on ice, immediately refrigerated upon leaving the field, and transported to the Salisbury Animal Health Diagnostic Lab (Maryland Department of Agriculture, Salisbury, Maryland, USA) or to the Frederick Animal Health Diagnostic Lab (Maryland Department of Agriculture, Frederick, Maryland, USA) for aerobic bacterial culture on blood agar plates. To harmonize procedures between the two laboratories, the microbiologists processing the cultures were in direct communication and used identical culture and organism identification protocols. A Gram stain and catalase test were conducted for all colonies that were growing. Aerobic bacteria were identified to genus and species based on morphology, staining characteristics, and biochemical characteristics based on standard American Society for Microbiology techniques (47, 48).

### Statewide study

Based on prior studies by our group and anecdotal evidence that IASM across Maryland differs regionally, and is more prevalent on the Eastern Shore, we hypothesized that habitat characteristics would impact the presence of *A. pyogenes* on deer. To test this we sought to establish the prevalence of *A. pyogenes* relative to habitat characteristics by sampling from six regions across Maryland. These regions approximately follow physiogeographic provinces specified by the Maryland Geological Survey (MGS), but sampled animals could only be identified to county of origin. Therefore, when county boundaries overlapped provinces, the entire county was assigned to the region that included the majority of the county land mass. Similarly, we divided the MGS**-**designated Blue Ridge province into the adjacent Ridge and Valley province and Piedmont Plateau province because of sampling difficulties. The westernmost Appalachian province was characterized by gently folded bedrock of shale, siltstone, and sandstone. The second westernmost Ridge and Valley province was characterized by poor soils and deeply folded sedimentary, shale, or sandstone bedrock. The central Piedmont Plateau province largely comprised eroded rocks of volcanic origin. The Coastal Plain regions of the east, the Western Shore, and the Upper Eastern and Lower Eastern shores were characterized by fertile soils and abundant ground water (49).

Data for this study were collected from October 18, 2010, to December 12, 2010. In each of the six regions, head and nasal swabs were collected from a total of 234 hunter-killed male deer (Appalachian N=50; Ridge and Valley N=21; Piedmont N=50; Western Shore N=33; Lower Eastern Shore N=32; Upper Eastern Shore N=48). The antlered:antlerless ratio (fawn males are included in antlerless numbers) in the statewide harvest was 1:2.2 and 1:1.96 in 2009 and 2010, respectively.

### White-tailed deer management study

To study the hypothesis that deer management practices may impact the prevalence of *A. pyogenes* on the Upper Eastern Shore region of Maryland, we collected head and nasal swabs from (N=113) hunter**-**killed male and female deer data from two properties with similar habitat characteristics, only one of which has documented history of IASM (18). The QDM property was 1,300 ha on Maryland's Eastern shore comprising 50% forest with non-alluvial swamps, 20% cropland, 13% fallow fields, with the remaining 17% comprising impoundments and other managed wildlife habitat (18, 50). The QDM property has been managed since 1994 under QDM, which fosters a balanced sex ratio and an older age structure among male deer (51). On the QDM property, white**-**tailed deer had been hunted annually with the harvest of males limited to individuals with antler spreads wider than ear tips (i.e. 2.5+ years old). Harvest male:female sex ratio was 1:3.4 and 1:2.5 for 2009 and 2010, respectively. Previous research identified IASM as a cause of 35% of mortality among adult males in the QDM population, and *A. pyogenes* had been cultured from live-captured adult males at this site (18). Flora of females and younger-age-class animals had not previously been evaluated.

The traditional deer management (TDM) property, which did not employ sex- or age-based harvest restrictions resulting in a young age structure among males and a heavily female-skewed sex ratio, was 925 ha comprising 37.6% marshland, 37.3% forest, 17.6% cropland, with the remainder being grassland, moist soil, waterwater, and developed area. The TDM property is located approximately 12.4 km from the QDM property on the same shoreline.. The white**-**tailed deer population had been hunted annually with no age or sex restrictions. Male:female sex ratios in recent harvests on the TDM property were 1.3:1 and 2.25:1 for 2009 and 2010, respectively.

### Neonate study

To examine the hypothesis of early (*in utero* or immediately postpartum) bacterial colonization of deer, head and nasal swabs from 11 neonates at the QDM property were collected June 5**–**8, 2009. The neonates were restrained manually for collection of head and nasal cultures. Survey sampling methods prevented collection of cultures from dams.

### Data analysis

For the statewide study, we evaluated the effect of region on whether deer carried *A. pyogenes* using Pearson's chi-square test with Yates’ continuity correction conducted in Program R (Version 2.9.1, http://cran.r-project.org, accessed April 25, 2009). For the white-tailed deer management study, we tested whether site, age class, and sex predisposed animals to carrying *A. pyogenes* using binary logistic regression with presence of *A. pyogenes* as the dependent variable, and site, age class, and sex as independent variables. Analyses were conducted in SYSTAT 13 (Systat Software, Chicago, Illinois, USA), and alpha was set at *P* ≤ 0.05.

## Results

### Statewide study

Prevalences of *A. pyogenes* and other bacteria across Maryland physiogeographic regions are summarized in Table 1 and Table 2. Physiogeographic region was a significant predictor of *A. pyogenes* presence for nasal samples (chi**-**square=111.684, df=1, *P*<0.001) and head samples (chi**-**square=74.932, df=1, *P*<0.001). We did not detect *A. pyogenes* on deer in three of the six physiogeographic regions studied. On the Lower Eastern Shore, we cultured *A. pyogenes* from only one (3%) nasal swab of 32 deer sampled and none of the head swabs. Similarly, on the Western Shore only one (3%) head swab and no nasal swab cultures included *A. pyogenes* out of 33 animals sampled. The Upper Eastern Shore was the only region where *A. pyogenes* was common; 45% (22/48) and 66% (32/48) of the antler and nasal swabs tested positive, respectively.

**Table 1:** Percent of white-tailed deer carrying *A. pyogenes* by region and swab type in Maryland, USA, 2010

	Head			Nasal		
		
Region	*n*	Mean isolates (range)	*A. pyogenes*%	*n*	Mean isolates (range)	*A. pyogenes*%
Appalachian	47	2.30 (1–6)	0	48	2.35 (1–4)	0
Ridge and Valley	18	1.33 (1–3)	0	19	1.95 (1–3)	0
Piedmont	49	3.06 (1–9)	0	48	3.17 (1–8)	0
Western Shore	24	1.35 (1–3)	0	23	1.48 (1–6)	4
Lower Eastern Shore	33	2.60 (1–5)	3	24	2.27 (1–4)	0
Upper Eastern Shore	44	4.05 (2–8)	45	41	3.80 (1–7)	66

**Table 2:** Bacteria carried by white-tailed deer (nasal%, head%) by region in Maryland, USA, 2010

Genus	Appalachian	Ridge and Valley	Piedmont	Western Shore	Lower Eastern Shore	Upper Eastern Shore
*Acinetobacter*	13, 26	11, 22	19, 37	0, 0	13, 30	2, 7
*Aerococcus*	0, 0	5, 0	0, 0	0, 0	0, 0	0, 0
*Aeromonas*	0, 0	5, 0	8, 0	0, 0	0, 0	0, 0
*Arcanobacterium*	0, 0	0, 0	0, 0	4, 0	0, 3	66, 45
*Bacillus*	25, 4	21, 0	56, 14	13, 8	13, 12	51, 61
*Chryseobacterium*	0, 0	16, 11	0, 0	0, 0	0, 0	0, 0
*Chryseomonas*	0, 0	0, 0	0, 0	0, 0	0, 0	24, 16
*Corynebacterium*	0, 0	0, 0	0, 0	0, 0	3, 4	5, 5
*Enterobacter*	0, 0	5, 6	0, 0	0, 0	0, 0	0, 2
*Enterococcus*	13, 4	32, 0	33, 12	9, 0	46, 18	2, 7
*Escherichia*	10, 0	6, 0	4, 2	0, 8	0, 0	32, 27
*Klebsiella*	2, 2	5, 0	0, 0	4, 0	0, 0	0, 0
*Kocuria*	0, 0	5, 0	0, 0	0, 0	0, 0	0, 0
*Mannheimia*	0, 0	0, 0	2, 0	0, 0	0, 0	0, 0
*Micrococcus*	0, 0	0, 0	0, 0	0, 0	0, 0	10, 0
*Moraxella*	15, 23	5, 0	44, 47	0, 0	38, 42	0, 2
*Pantoea*	17, 36	11, 17	48, 65	0, 4	17, 55	27, 30
*Pasteurella*	0, 0	0, 0	0, 0	0, 0	0, 0	7, 9
*Pectobacterium*	0, 0	47, 17	0, 0	0, 0	0, 0	0, 0
*Providencia*	0, 0	0, 0	0, 0	0, 0	0, 0	2, 0
*Pseudomonas*	42, 40	11, 6	50, 50	26, 29	54, 42	10, 9
*Serratia*	0, 0	0, 0	0, 0	0, 0	0, 0	5, 7
*Staphylococcus*	63, 64	11, 67	25, 35	52, 71	38, 61	66, 91
*Streptococcus*	10, 0	0, 0	0, 0	17, 0	4, 3	0, 0

### White-tailed deer management study

We cultured bacteria from 55 animals (33 females, 22 males) at the QDM property and 58 animals (21 females, 37 males) at the TDM property. The mean number of bacterial species isolated from the QDM property was 2.6 per nasal culture (range 1**–**4) and 3.0 per antler culture (range 1**–**5). Overall, 78% of animals sampled on the QDM property carried *A. pyogenes* on at least one swab; 54% of head swabs contained *A. pyogenes*, and 79% of nasal swabs carried *A. pyogenes*. The mean number of bacterial species isolated from the TDM property swabs was 3.6 per nasal culture (range 1**–**7) and 4.0 per head culture (range 1**–**8, Table 3, Table 4). Overall, 95% of the TDM property animals were positive for *A. pyogenes* on at least one swab; 84% of nasal swab cultures contained *A. pyogenes*, and 65% of head swab cultures contained *A. pyogenes*.

**Table 3:** Percent of white-tailed deer carrying *A. pyogenes* by sex and sample site on the Upper Eastern Shore in Maryland, USA, 2010

Site	Sex	*A. pyogenes*%	Nasal% only	Head% only	Head and nasal%
QDM property	F	77	26	0	52
QDM property	M	82	27	9	45
Total		78			
TDM property	F	100	19	5	76
TDM property	M	92	40	19	35
Total		95			

**Table 4:** Bacteria carried by white-tailed deer (nasal%, head%) at two properties in Maryland, USA, 2010

Genus	TDM property	QDM property
*Escherichia*	30, 23	17, 37
*Staphylococcus*	66, 81	37, 48
*Bacillus*	57, 75	23, 19
*Pantoea*	27, 25	40, 38
*Pseudomonas*	7, 4	25, 27
*Streptococcus*	0, 0	2, 0
*Proteus*	4, 0	0, 2
*Arcanobacterium*	84, 65	79, 56
*Corynebacterium*	4, 9	2, 12
*Chryseomonas*	23, 0	15, 0
*Micrococcus*	9, 0	0, 0
*Serratia*	4, 7	2, 4
*Penicillium*	9, 2	0, 0
*Providencia*	2, 0	0, 0
*Pasteurella*	11, 7	0, 2
*Mucor*	0, 2	0, 2
*Flavimonas*	2, 4	2, 2
*Enterobacter*	0, 2	4, 0
*Edwardsiella*	0, 0	2, 2
*Klebsiella*	2, 0	0, 2
*Acintobacter*	0, 2	0, 0
*Enterococcus*	0, 5	0, 19

**Fig. 2:**
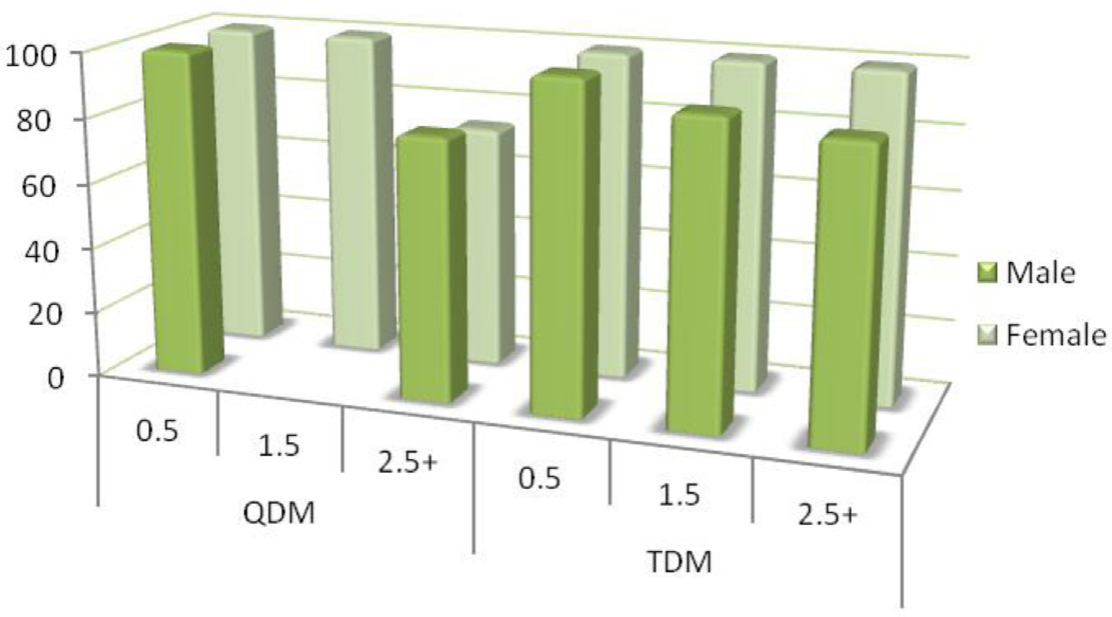
*A. pyogenes* percentage by sex, age class (0.5, 1.5, and 2.5+ years), and property on the Upper Eastern Shore, Maryland, USA.

Prevalence of *A. pyogenes* on hunter-killed deer was similar between the QDM and TDM properties for head (*P*=0.35) and nasal samples (*P*=0.34, Table 5). Similarly, the prevalence of *A. pyogenes* **-**positive cultures was similar between the sexes for head (*P*=0.13) and nasal samples (*P*=0.14) and across age classes for head (*P*=0.20) and nasal samples (*P*=0.99, Table 5, Fig. 2).

**Table 5:** Binary logistic regression for occurrence of *A. pyogenes* on white-tailed deer on the Upper Eastern Shore of Maryland, USA, 2010

						95% confidence interval	
	
Sample	Parameter	Estimate	Standard error	*Z*	*P*	Lower	Upper
Head	Site	−0.404	0.430	−0.939	0.348	−1.246	0.439
	Age	0.386	0.303	1.273	0.203	−0.209	0.981
	Sex	0.632	0.420	1.505	0.132	−0.191	1.455
							
Nasal	Site	0.514	0.540	0.953	0.340	−0.543	1.572
	Age	0.003	0.366	0.009	0.993	−0.713	0.720
	Sex	0.785	0.533	1.473	0.141	−0.259	1.829

### Neonate study

Eleven neonates were sampled on the QDM Property. *A. pyogenes* was not cultured from nasal or head swabs of any of these animals.

## Discussion

There is a tendency for health research to focus on highly communicable zoonotic diseases with devastating impacts on human patients (ebola, anthrax, bovine spongiform encephalopathy, and rabies), and in recent times, a particular emphasis has been placed on what are termed emerging diseases. However, less dramatic and more common zoonoses can have serious economic and environmental impacts. Endemic, chronic infections with a range of disease manifestations can impact one health in ways that may go unrecognized, despite the potential for control through relatively basic means. The concept of ‘emerging’ is particularly complex because recognition of a disease can be affected by observer and diagnostic effort with increasing prevalence simply reflecting greater awareness by the health community. *A. pyogenes* fits the basic definition of zoonosis from *Stedman's Medical Dictionary* as ‘an infection or infestation shared in nature by humans and other animals (52).’ It is carried by economically important wild and domestic animal species with high potential for close human contact and can cause disease in humans (17, 30, 34). The long recognition of *A. pyogenes* as a zoonosis argues against assigning it status as an emerging disease on the basis of definitions focused on recent identification of the pathogen, but because it is a zoonotic pathogen that has likely been under reported and misdiagnosed in humans, the term is not entirely unwarranted. The geographic differences in prevalence of *A. pyogenes* detected in our study suggest the disease may be considered ‘emerging’ in the sense of locality, regional environmental conditions, and the broadening of reservoir potential, including deer.

It is challenging to separate environmental and host factors in the epidemiology of disease occurrence. Our environmental study, limited to the six regions of Maryland, showed that site was an important factor in predicting whether *A. pyogenes* was carried by deer. Our design using two different state laboratories to process samples for logistical purposes would have been strengthened with the inclusion of positive controls created with laboratory strains of the organism, but we are confident of the results. Colony growth patterns and colony censuses on plates were similar between laboratories, and each laboratory positively identified *A. pyogenes* in submitted samples. In interpreting data, chi**-**square *P* **-**values can be problematic when a data set includes observed values of less than 5, but our results support the hypothesis that region plays a role in distribution of *A. pyogenes* in deer, and the *P* **-**values are so small that even serving as an approximation, they are highly significant. A broader variety of habitat types or a more fine-scale approach could have shown more profound differences. Our results indicate that environment plays a role in whether *A. pyogenes* is present.

*A. pyogenes* is considered commensal in domestic livestock species present across the state, but this pattern is not well documented for Maryland, nor is this the pattern of infection we observed in deer. Our results indicate the bacterium is not routinely carried by deer in most of Maryland, lending support to the notion that environmental conditions play a key role in *A. pyogenes* persistence.

Although the constellation of pathology compatible with IASM has been observed in regions of Maryland where we did not recover *A. pyogenes*, reports have anecdotally been concentrated on the Eastern Shore, which includes the region of high prevalence in our study (Eyler, Maryland Department of Natural Resources, personal communication). In addition, the clinical and gross pathological definitions of IASM are not sufficiently developed to reliably distinguish gross lesions associated with any given bacterium, and IASM occurs in the absence of *A. pyogenes*. Therefore, it is important that studies of IASM include careful bacteriological sampling to identify which bacteria are associated with the lesions. Other bacterial genera (i.e. *Staphylococcus*, *Pseudomonas*) that have been isolated from pyogenic cerebral lesions in white**-**tailed deer (17) are abundant and were recovered from more than 50% of animals sampled in every region of Maryland. The simple identification of an IASM**-**compatible lesion on a deer should not be considered a marker for *A. pyogenes* infection.

Similarly, presence of *A. pyogenes* in a white**-**tailed deer is not sufficient for IASM to develop. We detected similar prevalences of *A. pyogenes* on the QDM and TDM properties, but IASM has not been documented at the TDM Property, while IASM has become increasingly common at the QDM Property (18). It is likely that increased vigilance on the QDM property contributes to this disparity, but our results indicate that bacterial presence, sex ratio, and age structure may interact to help drive IASM prevalence on the Upper Eastern Shore. Therefore, deer management appears to play an important role in the extent to which IASM develops in populations where *A. pyogenes* is present at high levels. Conversely, the presence of IASM in a given population, as seen in our QDM population, may be an indication that *A. pyogenes* could be present, and humans and livestock who come into contact with deer may be exposed to the pathogen.

Our Upper Eastern Shore data indicate that *A. pyogenes* is endemic to that region and may even play a commensal role in some white**-**tailed deer populations. Although IASM is typically associated with adult male deer, the majority of deer sampled at both sites carried *A. pyogenes* in nasal passages, on heads, or both, regardless of sex or age. Management approach did not affect prevalence of *A. pyogenes*, providing further evidence that the bacterium could play a commensal role in deer populations on the Upper Eastern Shore and possibly similar habitats elsewhere. The question of maintenance of the pathogen remains open to further study.

We did not detect *A. pyogenes* in Upper Eastern Shore neonates, suggesting that neonates acquire the bacteria sometime after birth, presumably either through contact with their dams or environmental exposure sometime after the perinatal period examined in our study. Many of the neonates in our study were sampled within hours of birth, although some had been alive for several days. Even though these older fawns presumably could have been exposed to *A. pyogenes* carried by their dams through grooming interactions or in the environment, cultures of the fawns were negative. In contrast, most Upper Eastern Shore fawns sampled during the fall harvest (defined as the 0.5**–**year**-**old age class) tested positive for *A. pyogenes*, which indicates neonates eventually acquire *A. pyogenes* sometime during their first 6 months of life. A longer duration study of fawn flora identifying when fawns are colonized by *A. pyogenes* would help to clarify the role of deer social interactions, cross**-**species interactions, and environmental exposure in the acquisition of *A. pyogenes*.

If *A. pyogenes* is endemic to the Upper Eastern Shore of Maryland, it is worth considering the effect deer dispersal may have on distribution of the bacteria. It remains unclear how deer acquire *A. pyogenes* and to what extent carrying the bacteria predisposes them to developing IASM, but if intraspecific interactions are involved, dispersal may be an important factor. In our statewide survey, the only other regions where we detected *A. pyogenes*, the Lower Eastern Shore and the Western Shore, are adjacent to the Upper Eastern Shore. Future work could continue to monitor prevalence of *A. pyogenes* in deer and the occurrence of IASM in these areas while expanding surveillance to adjacent sites in Delaware and Pennsylvania.

**Fig. 3:**
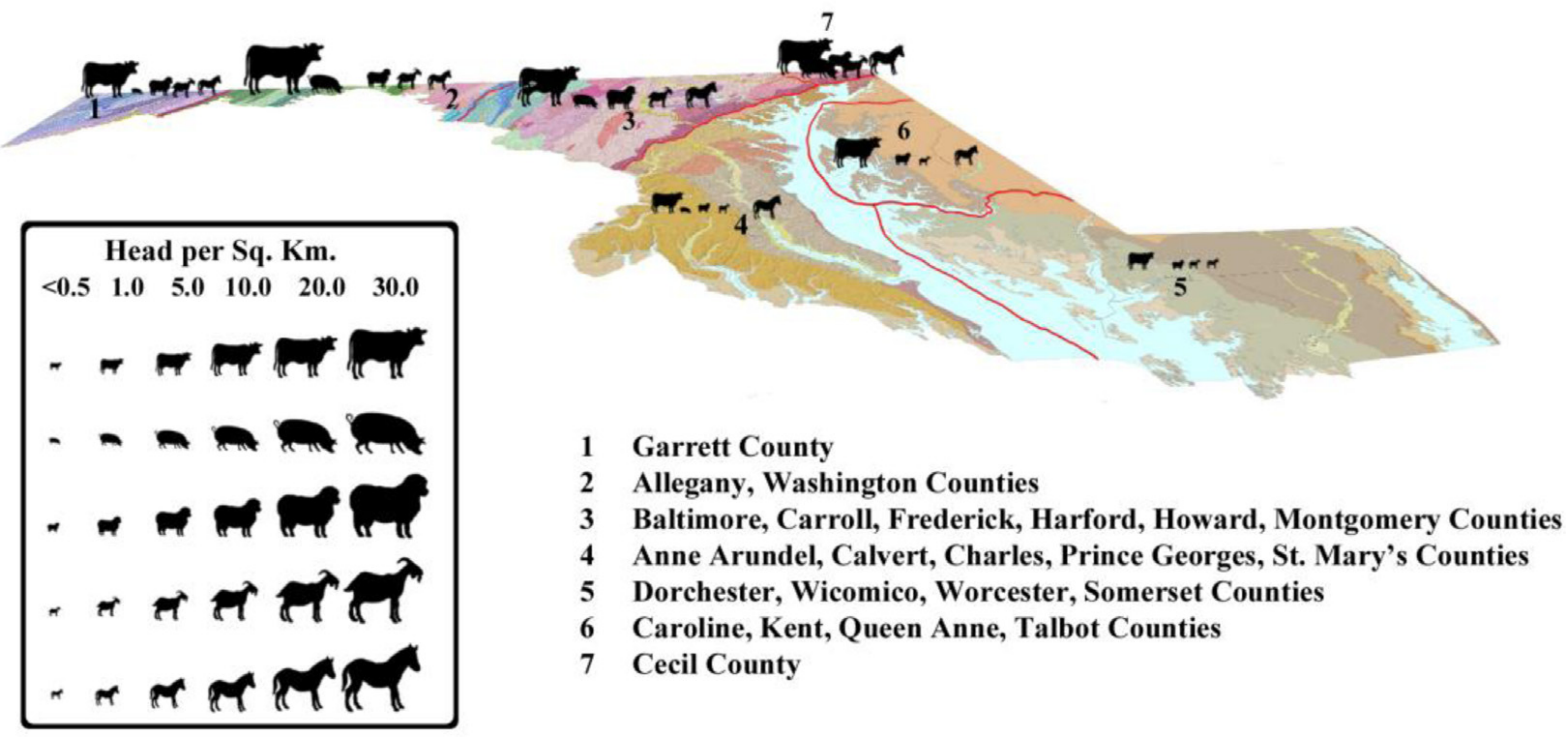
Livestock density by physiogeographic region in Maryland, USA.

The long**-**accepted view of *A. pyogenes* as a normal commensal organism associated with domestic production animals suggests the possibility that feral and/or production livestock could serve as reservoirs in endemic areas. Further, the environmental contamination with *A. pyogenes* by livestock, and/or livestock–deer interactions could be important in the maintenance of the disease in an accommodating environment. Fig. 3 presents relative livestock abundance in each of the six regions based on annual Maryland livestock inventory data from 2011 (53). The agriculture profile of the Upper Eastern Shore is primarily large**-**scale crop farming characterized by low livestock concentrations. The region ranks fourth of the six regions we sampled in cattle and sheep density and last in swine and goat density (53). This profile suggests that the presence of farm production animals alone may not explain the high prevalence of *A. pyogenes* in the Upper Eastern Shore. It may be important to examine the density of feral livestock and the potential for white**-**tailed deer or other wildlife species to play key roles in the maintenance of endemic *A. pyogenes*. The low observed prevalence of IASM coinciding with a high prevalence of *A. pyogenes* at the TDM property supports the potential role of white**-**tailed deer under traditional management to serve as a potential maintenance reservoir of *A. pyogenes* without any outward signs indicating that they are carrying the pathogen.

The framework of one health is useful for considering the broader implications of white**-**tailed deer, *A. pyogenes*, and IASM. Humans are not as far removed from white**-**tailed deer as they may perceive themselves to be. The seasonal close contact experienced by active deer hunters is a key point of one health intersection. Hunters could be educated about the identification of IASM lesions and the importance of proper sanitation and hygiene when handling a deer carcass (24, 24, 30). Many other intersections occur, primarily because the white**-**tailed deer thrives in the presence of humans and is not generally considered a threatening wildlife presence. The crepuscular, edge**-**occupying habits of white**-**tailed deer, and the perception they are non**-**aggressive, may cause the general public to underestimate their proximity and contact with these animals as well as that of companion and domestic animals. Providing information about *A. pyogenes* and possible clinical presentations and therapeutic options to medical and veterinary professionals in areas where high prevalence of *A. pyogenes* is detected in deer could improve disease recognition and outcome. The identification of a highly endemic area by our study offers the opportunity to better understand the actual health risk parameters, transmission routes, and environmental perturbations of disease occurrence, as well as potential of control measures in wildlife, humans, and domestic animals.
